# Photodegradation of Acid red 18 dye by BiOI/ZnO nanocomposite: A dataset

**DOI:** 10.1016/j.dib.2017.11.068

**Published:** 2017-11-24

**Authors:** Sahand Jorfi, Mohammad Javad Barkhordari, Mehdi Ahmadi, Neemat Jaafarzadeh, Azar Moustofi, Bahman Ramavandi

**Affiliations:** a Environmental Technologies Research Center, Ahvaz Jundishapur University of Medical Sciences, Ahvaz, Iran; b Department of Environmental Health Engineering, Ahvaz Jundishapur University of Medical Sciences, Ahvaz, Iran; c Department of Medicinal Chemistry, Ahvaz Jundishapur University of Medical Sciences, Ahvaz, Iran; d Department of Environmental Health Engineering, Bushehr University of Medical Sciences, Bushehr, Iran

**Keywords:** Photodegradation, Nanocomposite, BiOI/ZnO, Degradation, Dye, Acid red 18

## Abstract

Dyes are one of the most important existing pollutants in textile industrial wastewater. These compounds are often toxic, carcinogenic, and mutagenic to living organisms, chemically and photochemically stable, and non-biodegradable. Acid red 18 is one of the azo dyes that are currently used in the textile industries. Photocatalytic degradation offers a great potential as an advanced oxidation process, in this study photocatalytic degradation of Acid red 18 by using BiOI/ZnO nanocomposite was evaluated under visible light irradiation. The influence of most essential parameters such as pH and BiOI/ZnO dosage were studied for optimum conditions. The dye removal efficiency was 85.1% at optimum experimental conditions of pH of 7, and BiOI/ZnO dosage of 1.5 g/L. The data had a good agreement with pseudo first-order kinetic model. Thus, the BiOI/ZnO/UV is an efficient process for dye degradation.

**Specifications Table**

**Table:** 

Subject area	*Environmental engineering*
More specific subject area	*Advanced oxidation process*
Type of data	*Figure and table*
How data was acquired	– *A reactor (6 cm diameter*×*16 cm height) was equipped with five hallogen lamps (60* *W, Osram).* – *A given concentration of Acid red 18 (20–100 mg/L) was poured in the reactor.* – *A given dosage of BiOI/ZnO nanocomposite (0.5–3 g/L) was used as photo-catalyst.* – *Sample was taken after designated time (60 min) for Acid red 18. The residual concentration of dye was measured by a UV–vis spectrophotometer model V-530 at 505 nm (Jasco, Japan).*
Data format	*Analyzed data*
Experimental factors	*The effect of solution pH and BiOI/ZnO dosages was evaluated during the experiments of Acid red 18 degradation.*
Experimental features	*Acid red 18 degradation by BiOI/ZnO/UV process*
Data source location	*Environmental Technologies Research Center, Ahvaz Jundishapur University of Medical Sciences, Ahvaz, Iran, 31°19′13″N 48°40′09″E*
Data accessibility	*Data are present in this article only*

**Value of the data**

• A novel and promising photocatalyst of BiOI/ZnO nanocomposite was developed to photocatalysis of Acid red 18 from aqueous environment.
• This dataset would put forward a facile system of BiOI/ZnO/UV for oxidation of recalcitrant pollutants like azo dyes from wastewater.
• The data also showed that BiOI/ZnO/UV process is highly active in neutral media; this would be beneficial from economically point of view as most of the wastewaters have a neutral pH and no further material is required for pH adjusting.

## 1 Data

**Fig. 1:**
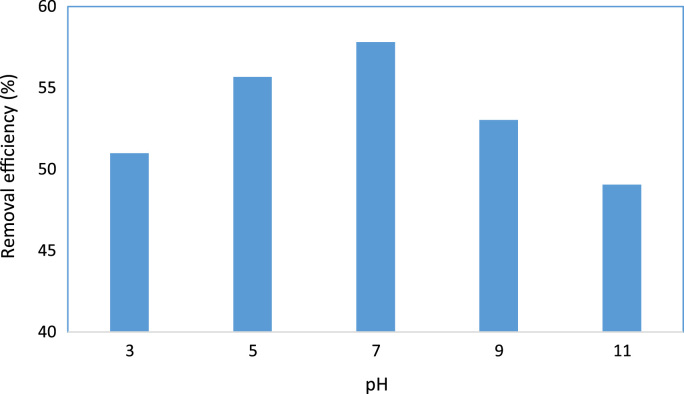
Effect of pH on Acid red 18 degradation by BiOI/ZnO/UV process (Acid red 18=69 mg/L, BiOI/ZnO=0.75 g/L, reaction time=60 min).

**Fig. 2:**
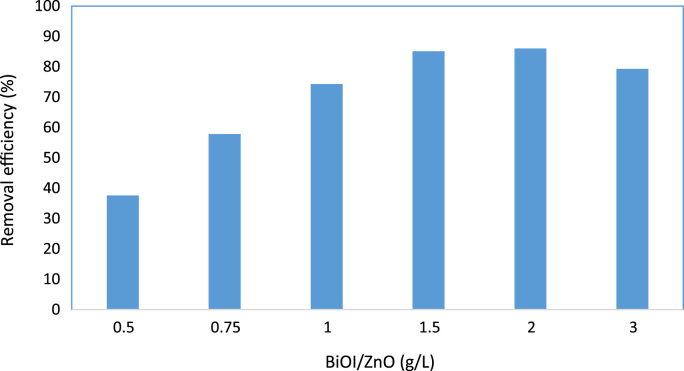
Effect of BiOI/ZnO dosage on Acid red 18 degradation by BiOI/ZnO /UV process (pH=7, Acid red 18=60 mg/L, reaction time=60 min).

Data presented in this paper described the effectiveness of BiOI/ZNO/UV process in Acid red 18 degradation. Fig. 1, Fig. 2 show the effect of pH and BiOI/ZNO dosage on the photo-catalytic degradation of Acid red 18, respectively. Also, Table 1 represents pseudo first-order kinetic model for Acid red 18 degradation by BIOI/ZnO /UV process.

**Table 1:** Pseudo first-order kinetic model for Acid red 18 degradation by BiOI/ZnO /UV process (BiOI/ZnO: 1.5 g/L, pH:7).

Dye concentration (mg/L)	Pseudo first order model	
	*R*^2^	*k* (min^−1^)
10	0.8314	0.0376
20	0.8617	0.0339
40	0.8511	0.0279
60	0.8419	0.0225
80	0.8404	0.0175
100	0.8615	0.0123

## 2 Experimental design, materials and methods

Acid red 18 used to determine the performance of the catalyst was bought from Alvan Sabet Co. Hamadan, Iran. All stock solutions were prepared using double-distilled water. Zinc nitrate (Zn(NO_3_)_2_.6H_2_O), potassium iodide (KI), bismuth nitrate (Bi(NO_3_)_3_·5H_2_O), sodium hydroxide (NaOH) and sulfuric acid (H_2_SO_4_) were provided from Fluka Co. ZnO/BiOI nanocomposite were synthesized by a facile chemical bath method at low temperature [1].

A glass reactor was used in this study and irradiations were carried out using five visible light halogen lamp (300 W, Osram). The distance between the halogen lamp and the Acid red 18 solution container was 10 cm. The reactor was filled with a 200 mL of defined concentration of dye and then the nanocomposite was added.

The temperature of the tested solution was maintained at 25±2°C. The solution pH was adjusted by means of 0.1 M H_2_SO_4_ or NaOH solutions. Samples were collected at regular intervals during irradiation and centrifuged before analysis. Acid red 18 concentration was determined using UV–Vis spectrophotometer (DR-5000) at *λ*_max_=505 nm and the degradation efficiency was calculated by Eq. (1):

(1) $$Degradation\;efficiency\left(\%\right)=\left(C_{0}–C_{t}\right)/C_{0}\times 100$$

where, the *C*_0_ and *C*_t_ are the initial and the residual Acid red 18 concentration, respectively [2], [3].

The kinetic parameters of zero, pseudo first and pseudo-second-order kinetic models including rate of BiOI/ZnO /UV process for dye degradation were determined by plotting *C*_t_ versus time, ln(*C*_0_/*C*_t_) versus time and 1/*C*_t_ versus time, respectively. The individual kinetic equations are reported as follows (Eq. (2) zero order, Eq. (3) pseudo first-order and Eq. (4) pseudo second-order [4], [5], [6], [7]).

(2) $$C_{t}=C_{0}-k_{0}t$$

(3) $$\ln\;C_{0}/C_{t}=k_{1}t$$

(4) $$1/C_{t}-1/C_{0}=k_{2}t$$

where, *t* is the reaction time (min) and *k*_n_ is the corresponding rate constants (*n*=0, 1 and 2).
